# Proteome and transcriptome analyses of wheat near isogenic lines identifies key proteins and genes of wheat bread quality

**DOI:** 10.1038/s41598-021-89140-4

**Published:** 2021-05-11

**Authors:** Liangjie Lv, Aiju Zhao, Yelun Zhang, Hui Li, Xiyong Chen

**Affiliations:** Institute of Cereal and Oil Crops, Hebei Academy of Agriculture and Forestry Sciences, Crop Genetics and Breeding Laboratory of Hebei, Shijiazhuang, China

**Keywords:** Agricultural genetics, Genomics, Plant breeding

## Abstract

The regulation of wheat protein quality is a highly complex biological process involving multiple metabolic pathways. To reveal new insights into the regulatory pathways of wheat glutenin synthesis, we used the grain-filling period wheat grains of the near-isogenic lines NIL-723 and NIL-1010, which have large differences in quality, to perform a combined transcriptome and proteome analysis. Compared with NIL-1010, NIL-723 had 1287 transcripts and 355 proteins with significantly different abundances. Certain key significantly enriched pathway were identified, and wheat quality was associated with alanine, aspartate and glutamate metabolism, nitrogen metabolism and alpha-linolenic acid metabolism. Differentially expressed proteins (DEPs) or Differentially expressed genes (DEGs) in amino acid synthesis pathways were upregulated primarily in the glycine (Gly), methionine (Met), threonine (Thr), glutamic acid (Glu), proline (proC), cysteine (Cys), and arginine (Arg) synthesis and downregulated in the tryptophan (trpE), leucine (leuC), citrulline (argE), and ornithine (argE) synthesis. Furthermore, to elucidate changes in glutenin in the grain synthesis pathway, we plotted a regulatory pathway map and found that DEGs and DEPs in ribosomes (RPL5) and the ER (HSPA5, HYOU1, PDIA3, PDIA1, Sec24, and Sec31) may play key roles in regulating glutenin synthesis. The transcriptional validation of some of the differentially expressed proteins through real-time quantitative PCR analysis further validated the transcriptome and proteomic results.

## Introduction

Wheat (*Triticum aestivum* L.) is one of the three most important crops worldwide. It is consumed by approximately 35% of the world’s population, the global output of wheat in 2019 was approximately 740 million tons (http://www.fao.org/worldfoodsituation/csdb/en/). Thus, wheat is a major crop in the diet of people worldwide^1^. Its flour can be processed into various foods, such as noodles, steamed buns, bread, biscuits, and cakes^2^. The flow characteristics of dough are important indicators reflecting the processes of kneading, bread forming, and dough fluffing^3^. Studies have reported a significant correlation between flow characteristics and baking performance^4^. Gluten is considered an essential ingredient that affects the properties of wheat dough and main source of viscoelasticity. Many studies have affirmed that the quality of wheat flour processing depends primarily on the effects of gliadins and glutenins on the flow characteristics of wheat dough^5,6^. Glutenins can form multimeric proteins that primarily affect the elasticity of dough and ultimately influence the processing quality of wheat flour^7^. Therefore, studies concerning the composition, structure, and function of glutenins have been central in the field of wheat quality improvement^3,5^.

Glutenins are classified into high molecular weight glutenin subunits (HMW-GSs) and low molecular weight glutenin subunits (LMW-GSs) on the basis of electrophoretic mobility^7^. HMW-GSs have been confirmed to be a determinative factor affecting dough behavior and wheat quality^6^ and are essential for measuring dough viscosity and elasticity during flour processing^8^. Although HMW-GSs account for only 8–10% of wheat grain protein, their allelic variation accounts for 4%–70% of the variation in wheat flour processing quality. HMW-GS is encoded by the *glu1* loci on the long arms of chromosomes 1A, 1B, and 1D. The allelic variation of the *glu1* locus is closely associated with gluten quality^8^. In theory, 6 HMW-GS genes can be expressed in hexaploid wheat, but due to gene silencing, usually only three (*1Bx*, *1Dx*, and *1Dy*) to five (*1Ax*, *1Bx*, *1By*, *1Dx*, and *1Dy*) are expressed^2^. Generally, the *1Bx*, *1Dx*, and *1Dy* coding genes are actively expressed, whereas the *1Ax* and *1By* coding genes, especially the *1Ay* subunit, are sometimes silenced in hexaploid wheat^9^. However, the Swedish W3879 hexaploid wheat line carries active *1Ax* and *1Ay* alleles, and Roy et al*.*^10^ introgressed the 1Ay21* gene into Australian wheat cultivar, Lincoln.

The structural characteristics of HMW-GSs in wheat dough during the baking process are believed to be associated with gluten polymers. The number and distribution of cysteine residues determine the formation of inter and intramolecular disulfide bonds^11^. Previous studies have argued that the length of the repetitive domains is positively correlated with their effects on the strength of wheat dough because the subunits of long repetitive domains can form more stable interactions through hydrogen bonding between chains, such as 1D × 5 subunits^2^. The *glu-a1* and *glu-d1* loci of HMW-GS have been affirmed to have a positive effect in enhancing the microstructure and agglomeration of the gluten matrix, thereby imparting the wheat dough with superior rheological properties^12–14^. Ravel et al*.*^15^ designed a series of 17 SNP markers that represent the most common alleles at each locus and which better capture the allelic diversity of the *glu-a1*, *glu-b1*, and *glu-d1* loci.

The influence of LMW-GS on flour processing quality has been studied for many years. Many recent studies have focused on understanding the effects of divergent LMW-GS alleles on the quality of bread wheat^16–18^. LMW-GSs (accounting for approximately 60% of total gluten) are a key factor in determining wheat processing end product quality and are more effective than HMW-GSs in some cases^19^. LMW-GSs are also encoded by the *glu-a3, glu-b3*, and *glu-d3* genes at the *glu3* locus on the short arms of homoeologous group 3 chromosomes, and there are 30–43 copies in the wheat genome^20,21^. Moreover, LMW-GSs are classified into LMW-m, LMW-s, and LMW-i types on the basis of the first N-terminal amino acid (methionine, serine, and isoleucine, respectively)^7,21,22^. In recent years, researchers have cloned and identified distinct LMW-GS alleles, including alleles 4, 3, and 7 of the *glu-a3*, *glu-b3*, and *glu-d3* loci in the Xiaoyan 54 wheat cultivar^23^; alleles 1, 5, and 7 in the Dayang cultivar^20,24^; and alleles 1, 2, and 6 of the Keumkang cultivar^21^. Zhang et al*.*^16^ reported that different LMW-GS alleles provide various degrees of strength and ductility wheat dough using Aroona near-isogenic lines. HMW-GS and LMW-GS alleles in distinct bread wheat varieties determine bread quality. Based on quality scores, studies have been conducted to improve the ductility and elasticity of wheat dough and the quality of final products^25,26^.

With the development of DNA sequencing technology, transcriptomics and proteomics are increasingly employed in studies of plant quality to identify the overall patterns of change in research subjects. Although transcriptomics and alternative splicing studies have provided considerable assistance in studies of wheat quality, proteins are the ultimate manifestation of many biological processes and cell functions, and the process from mRNA to protein also includes processes such as RNA alternative splicing and mRNA translation. In addition, studies regarding the transcriptome do not focus on processes that reveal physiological changes most directly. Hence, directly studying proteomic changes in plants, in conjunction with the transcriptome, is necessary^27,28^. With the development of proteomics research technology, isobaric tags for relative and absolute quantitation (iTRAQ) are becoming widely used in plant proteomics research. Zhong et al*.*^29^ utilized iTRAQ technology to test the effect of nitrogen topdressing timing on changes in wheat gluten quality and grain hardness and identified 591 proteins in 17 functional categories. Nine γ-gliadins or HMW glutenin subunit DEPs associated with nitrogen topdressing timing were found. Galland et al*.*^30^ employed transcriptomics and proteomics to analyze changes in starch biosynthetic enzymes and seed endosperm storage proteins in the late stages of rice seed development, demonstrating the value of multi-omics in the study of rice seed nutritional quality. Thus, an integrative analysis of transcriptomics and proteomics were used in the present study to further investigate the synthetic mechanisms and regulatory pathways in wheat bread quality.

## Results

### Characterization of NIL-723 and NIL-1010

We identified the quality between NIL-723 and NIL-1010 as near-isogenic lines in the quality testing center of crop varieties in Hebei Province, and found that there were only differences in quality traits. (Table 1), and other agronomic traits were similar. In this study, the protein, water absorption, dough development time, extension area and sedimentation value of NIL-723 were slightly higher compared with NIL-1010, but not statistically significant. The dough stability time and the maximum resistance were significantly higher than those of NIL-1010 (*P* < 0.05). Previous studies have elucidated that the content and type of glutenin have a greater impact on the quality of wheat^6^, while the main differences between the near isogenic lines in this study lie in the dough stability time and the maximum tensile resistance.

**Table 1:** Quality measurement index of NIL-723 and NIL-1010.

Material	Protein (%)	Water absorption (%)	Dough development time (min)	Dough stability time (min)	Extension area (cm^2^)	Maximum resistance (EU)	Sedimentation value (ml)	Wet gluten (%)
NIL-1010	14.31	60.65	4.9	3.9	81	413	39.65	28.6
NIL-723	14.83	61.36	5.9	60.2**	114*	804**	41.15	28.1

*, **Represents significant difference at *P* < 0.05 and *P* < 0.01, respectively.

### Screening significant differential transcripts and proteins

**Figure 1:**
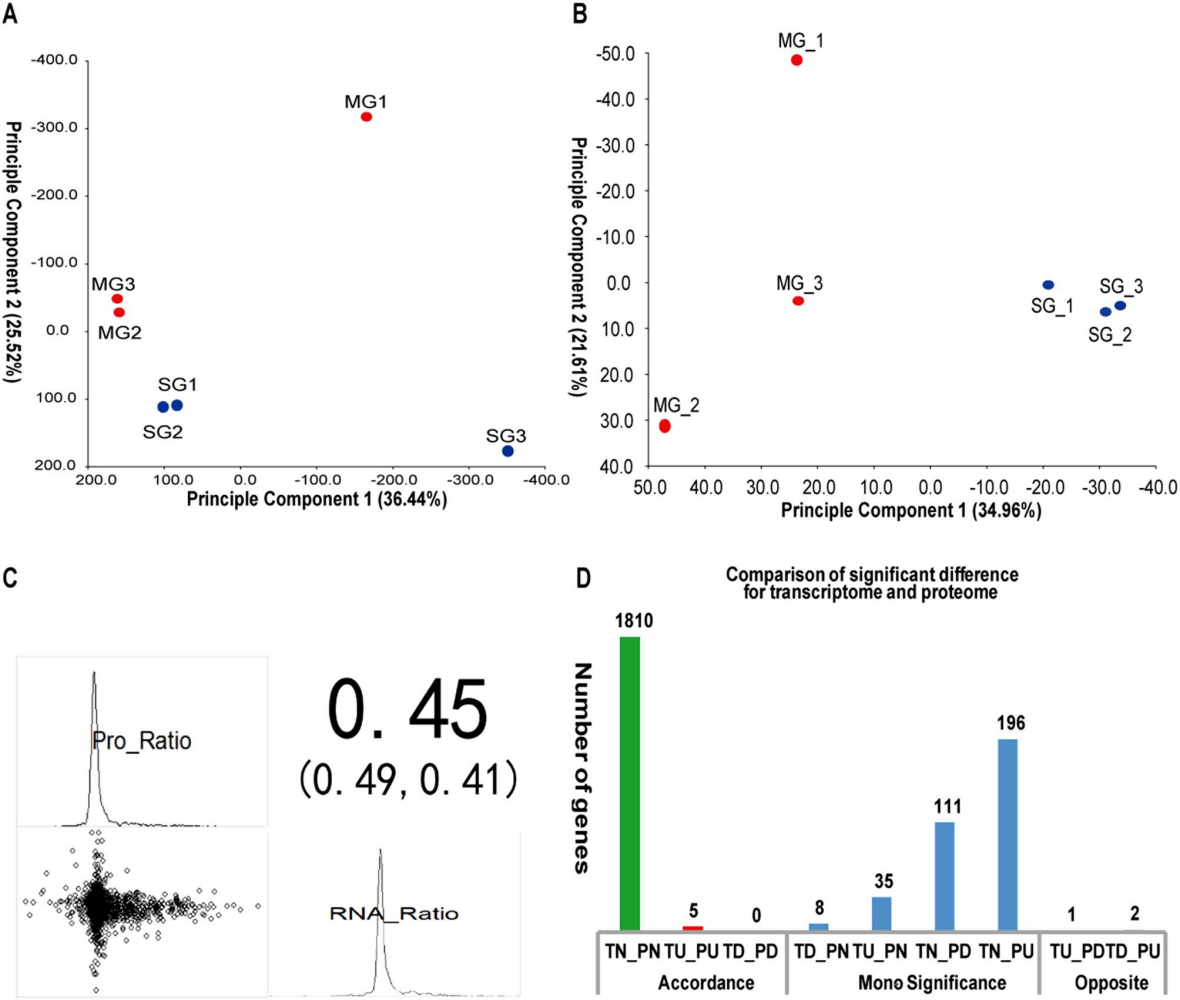
(**A**) Principal component analysis at the transcriptome level. SG-gene represents genes of NIL-723, MG-gene represents genes of NIL-1010, SG-protein represents proteins of NIL-723, MG-protein represents proteins of NIL-1010. (**B**) Principal component analysis at the proteome level. (**C**) The correlation analysis of abundance changes from transcriptome to proteome. Expression values were normalized by z-scoring, and the Pearson correlation coefficient was calculated for all genes with corresponding proteins identified in 723 and1010. (**D**) Comparison of significant differences between the transcriptome and proteome. T: transcript; P: protein species; N: no change; U: up-regulation; D: down-regulation. Accordance represents a consistent changing trend between the transcriptome and proteome; Mono significance represents mono-significant differences between the transcriptome and proteome; opposite represents that the transcriptome and proteome have opposite changing trends. plot generated using the ‘ggplot2’ packages in R^31,32^.

**Figure 2:**
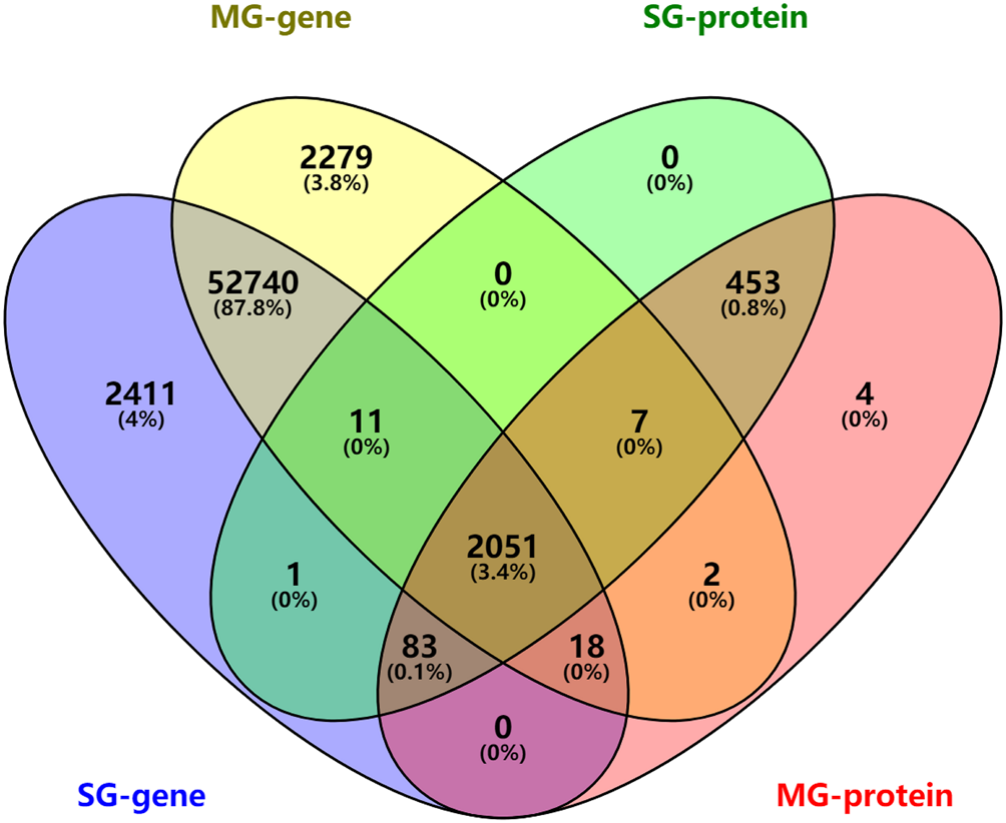
Venn diagram of transcriptome and proteome between NIL-723 and NIL-1010. The numbers in parentheses showed percentages concerning the total up-regulated and down-regulated. SG-gene represents genes of NIL-723, MG-gene represents genes of NIL-1010, SG-protein represents proteins of NIL-723, MG-protein represents proteins of NIL-1010. Veen diagram was generated usingInteractiVenn (http://www.interactivenn.net).

The quality analysis confirmed that the near-isogenic lines in this study differed primarily in stabilization time and maximum tensile resistance. Therefore, grains were collected at 25 days after flowering, and combined transcriptomic and proteomic analysis was used to study regulatory mechanisms affecting wheat quality. Through the high-throughput DNA sequencing and filtering of invalid data, 37,762,443 (11.33G) clean reads were obtained, 125,219 genes were aligned to the wheat genome (Table 2), and 57,315 genes in NIL-723 and 57,108 genes in NIL-1010 were detected in the transcriptome. Distinctions between materials determine functional specificity, and principal component analysis showed a clear separation between NIL-723 and NIL-1010 in the transcriptome (Fig. 1A) and proteome (Fig. 1B). A comparative analysis of transcriptome data was performed, which included 1287 DEGs with |log_2_ (fold change) |> 2, *p* < 0.05, of which 869 genes were downregulated and 418 were upregulated in NIL-727 in comparison to NIL-1010 (Table 2, (Table S1)). Label-free technology was used for the quantitative analysis of proteomic changes associated with wheat quality. A total of 13,411 unique peptide fragments were identified from the six samples (Table S2). A total of 2606 proteins from NIL-723 and 2618 proteins from NIL-1010 were detected in the proteome (Table 1), of which 124 proteins were downregulated and 231 proteins were upregulated. The intersection and union of the DEGs and differentially expressed proteins (DEPs) of the experimental materials were analyzed using Venny online software (http://bioinfogp.cnb.csic.es/tools/venny/index.html). A comparison between the two wheat lines showed that NIL-723 contained 2495 specific genes and 12 specific proteins and that NIL-1010 contained 2288 specific genes and 24 specific proteins (Fig. 2). Among them, one DEP detected in the proteome was the same as the protein encoded by DEG in NIL-723, and two DEPs were identical with the proteins encoded by DEGs in NIL-1010.

**Table 2:** Results of identification genes and quantitative proteins.

Gene	NIL-723 versus NIL-1010	Protein	NIL-723 versus NIL-1010
Clean reads	37,762,443	Polypeptide species identified	13,411
Identified genes	125,219	Identified proteins	2618
DEGs	1287	DEPs	355
Upregulated DEGs	418	Upregulated DEPs	231
Downregulated DEGs	869	Downregulated DEPs	124

### Dynamics of transcript-to-protein between NIL-723 and NIL-1010

Data regarding proteins and mRNA with the same changes in expression can be used to further confirm and explain certain crucial mechanisms in organisms. On the basis of protein identification and transcriptome sequencing results, threshold conditions were selected for screening-relevant DEGs and DEPs. Genes with total reads < 10 in the six samples were eliminated to ensure the accuracy of the transcriptome. Threshold conditions for DEGs were as follows: fold difference ≥ 2; threshold condition for DEPs: fold difference ≥ 1.2. The R program was used to study consistency concerning changes in the transcript abundance of the types of proteins. Expression values were normalized by z-scoring, and the Pearson correlation coefficient was calculated for all genes with corresponding proteins identified in 723 and1010. The results asserted that NIL-723 and NIL-1010 had similar changes in most transcripts and protein species (Fig. 1C, Table S3). 1815 members showed a consistent changing trend between the transcriptome and proteome, includes 5 members that were upregulated in both transcriptome and proteome; 350 members had mono-significant differences. Among them, 8 and 35 members were down-regulated and up-regulated in the transcriptome, but there was no significant difference in the proteome. Besides, 111 and 196 members were down-regulated and up-regulated in the proteome, respectively, but there was no significant difference in the transcriptome; 3 members were in opposite directions (Fig. 1D). These analyses indicated that gene expression does not fully represent the abundance of protein species and that there may be strong post-translational regulation during protein production^28^.

### GO and pathway analysis of genes in the transcriptome and proteome

**Figure 3:**
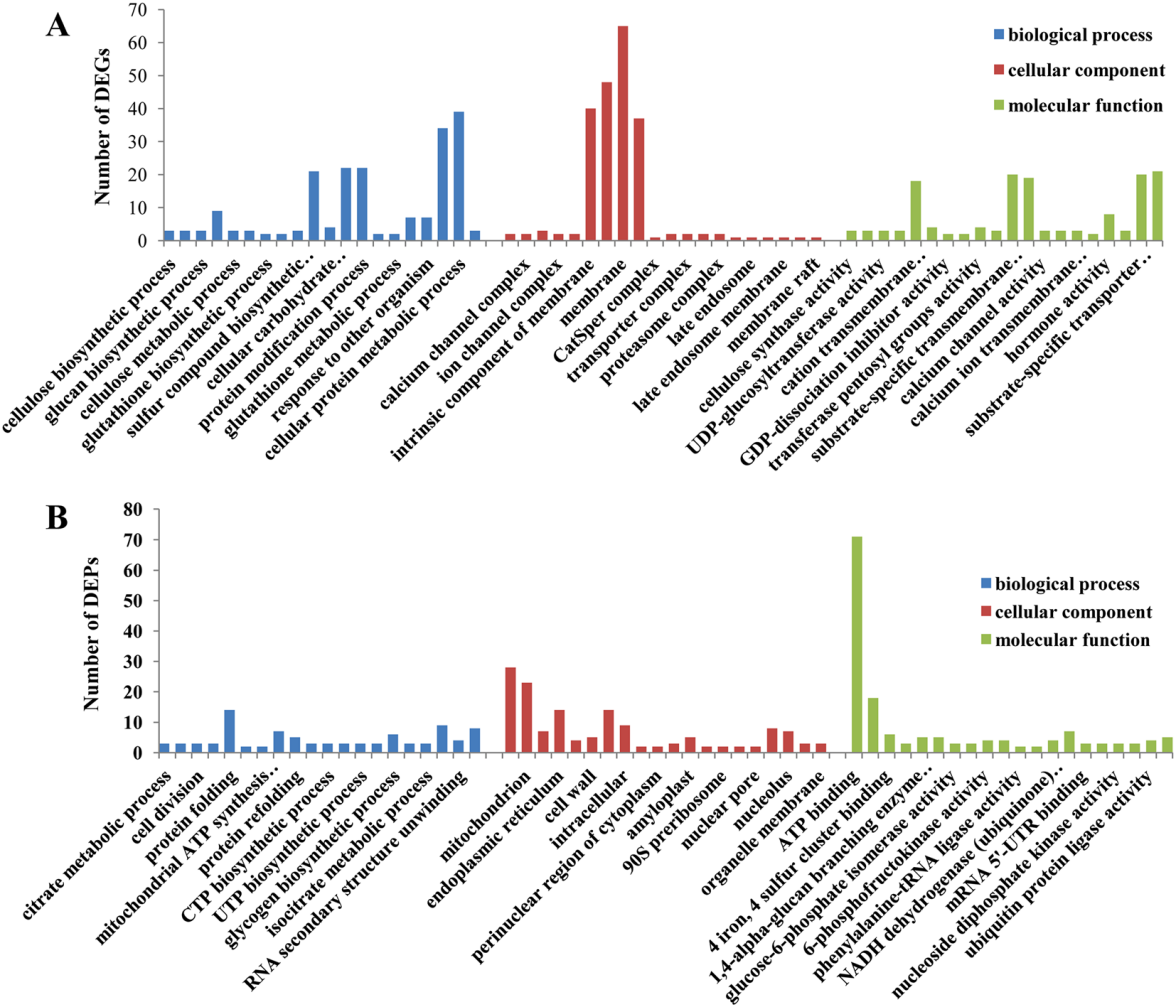
(**A**) GO categories for significantly differentially expressed genes in the transcriptome. (**B**) GO categories for significantly differentially abundant protein species in the proteome. Color panels highlight the three GO terms in this study, and the bar graph represents the unigene and protein number. plot generated using the ‘ggplot2’ packages in R^31,32^.

**Figure 4:**
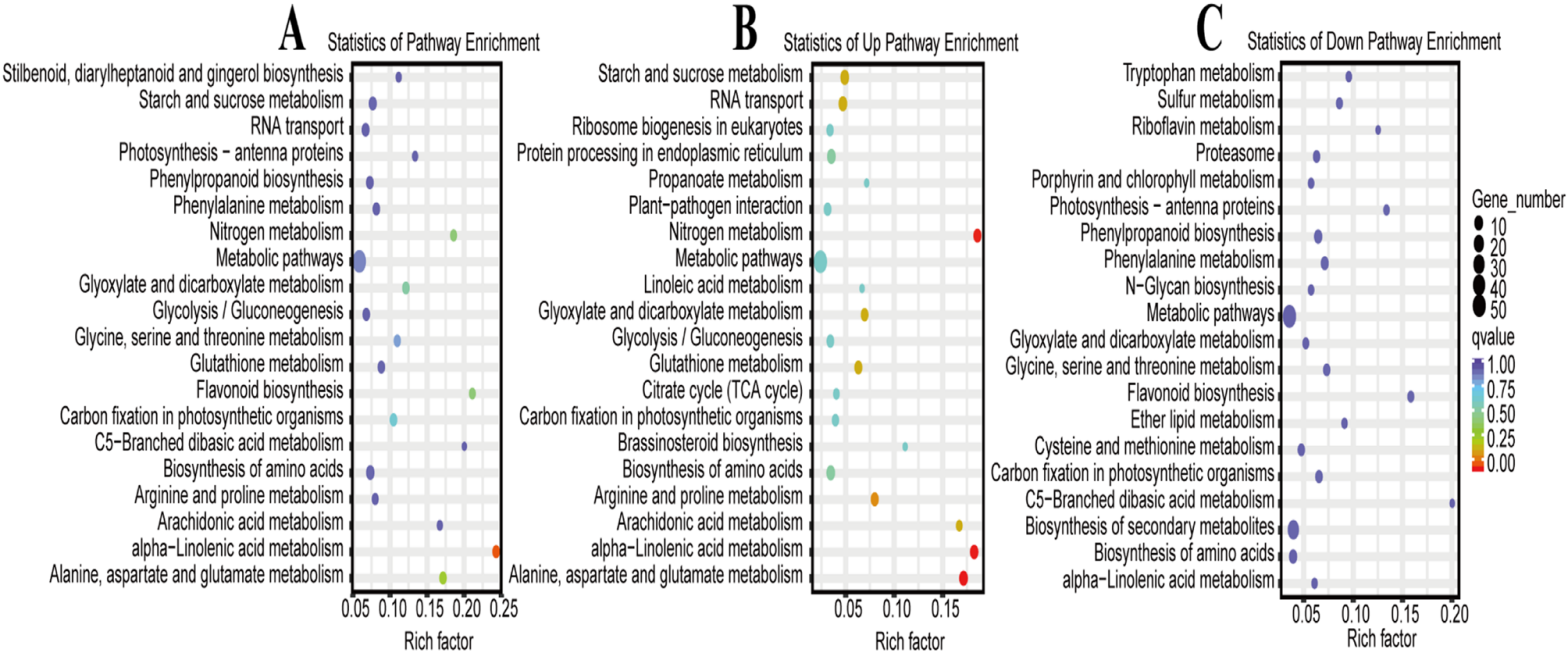
Gene pathway analysis of combined transcriptome and proteome analyses. (**A**) All genes, (**B**) Up-regulated genes, (**C**) Down-regulated genes. The sizes of the dots represent the gene numbers of each row, and *p* values were calculated from hypergeometric tests. plot generated using the ‘ggplot2’ packages in R^31,32^.

The GO functional annotation of DEGs was performed. These DEGs were categorized into three GO terms, namely, biological process, cellular component, and molecular function. Cellular protein metabolic processes accounted for the largest proportion among biological processes (Fig. 3A, Table S4). Membrane ranked first among cellular components, and transmembrane transporter activity was the largest category among molecular functions. These DEPs were also categorized into three GO terms (Fig. 3B, Table S5). Protein folding accounted for the largest proportion among biological processes. Chloroplast ranked first among cellular components, and ATP binding was the largest category among molecular functions. The KEGG analysis of consistent genes after combined transcriptomic and proteomic analysis revealed that most of the genes were enriched in alpha-linolenic acid metabolism (Q value < 0.05, Fig. 4A, Table S6). Significantly upregulated genes were mostly involved in the alanine, aspartate and glutamate metabolism, nitrogen metabolism and alpha-linolenic acid metabolism (Q value < 0.05, Fig. 4B, Table S7), however, significantly downregulated genes were not enriched into any pathway (Q value < 0.05, Fig. 4C, Table S8).

### Protein–protein interaction network

**Figure 5:**
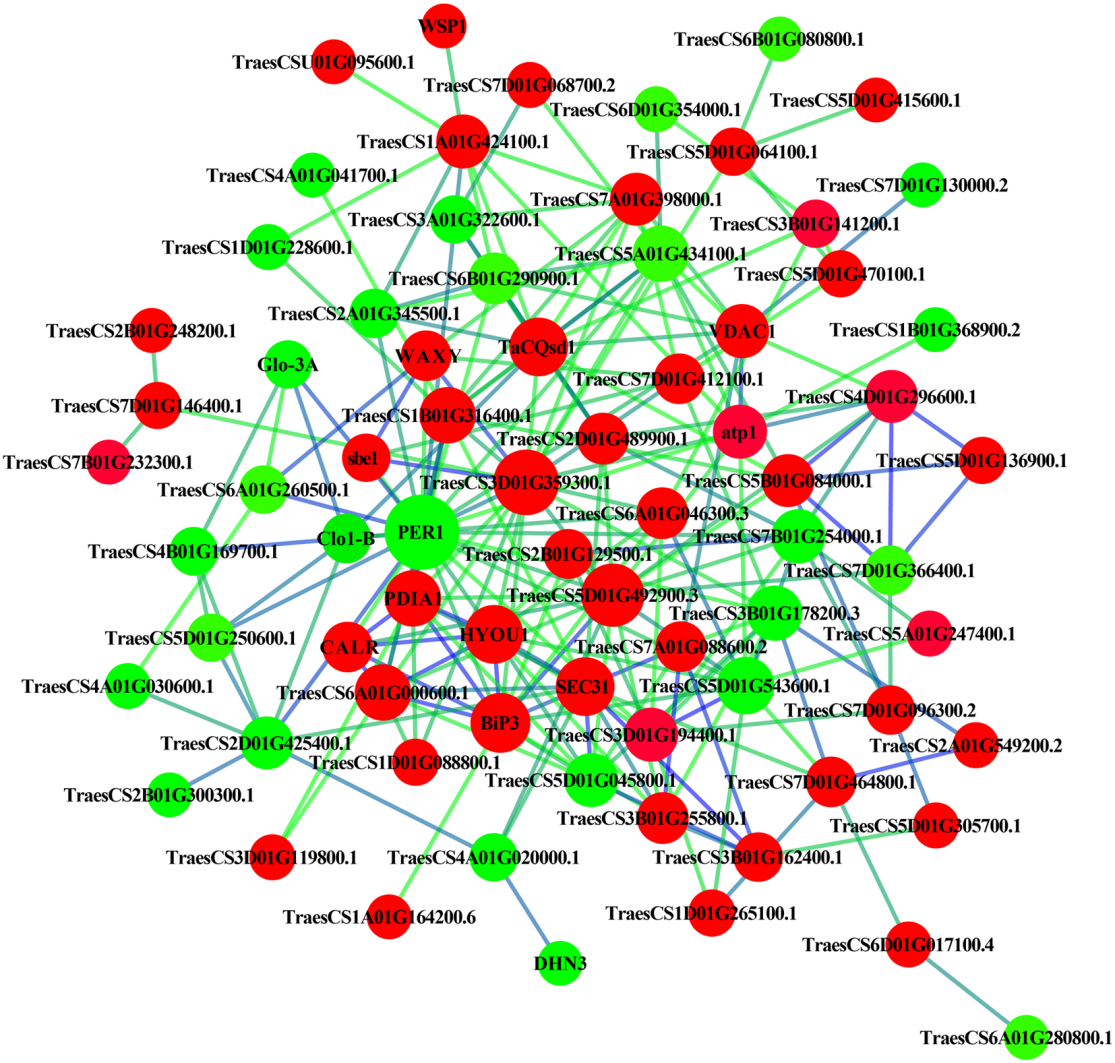
Protein–protein interaction network of protein species that are differentially accumulated between NIL-723 and NIL-1010. A green background indicates down-regulation of protein species, a red background indicates up-regulated protein species. The networks between NIL-723 and NIL-1010 were visualized with Cytoscape software (version 2.8.2, https://cytoscape.org/).

We constructed a protein–protein interaction network with the top 100 differential genes and proteins (Fig. 5, Table S9). In the network, a third of the significantly downregulated protein species and two-thirds of the significantly upregulated protein species were found to be related to the synthesis and assembly of starch and protein, suggesting that the excellent quality of NIL-723 may be affected by the synthesis and assembly of starch and protein, such as TraesCS6A01G000600.1 (PDIA3), TraesCS3B01G162400.1 (SEC13), TraesCS2D01G600900.1 (BiP3), and TraesCS2D01G546500.2 (CALR), and maybe involved in glutenin development. TraesCS7D01G535600.3 (sbe1), TraesCS7A01G070100.1 (WAXY), and TraesCS7D01G190100.1 are essential enzymes involved in the synthesis of starch, which also affects the structure of gluten. TraesCS4B01G017600.1 (Glo-3A), TraesCS7B01G489800.1, TraesCS4A01G067700.3, TraesCS3D01G119800.1 and TraesCS4D01G171800.1 were related to the synthesis of storage proteins.

The figure shows that the expressions of HYOU, BiP3, PDIA1, CLAR, Sec31, TraesCS5D01G492900.3, and TraesCS3D01G359300.1 were all upregulated and made greater contributions to protein interactions, suggesting that these proteins are associated with the excellent quality of NIL-723 (Fig. 5). PER1, Clo1-B, TraesCS5D01G045800.1, TraesCS5D01G543600.1, TraesCS3B01G178200.3, and TraesCS7B01G254000.1 were all downregulated and made larger contributions to protein interactions, suggesting that the downregulation of these proteins also affects wheat quality.

### Wheat quality-related DEGs and DEPs in the biosynthesis of amino acids

**Figure 6:**
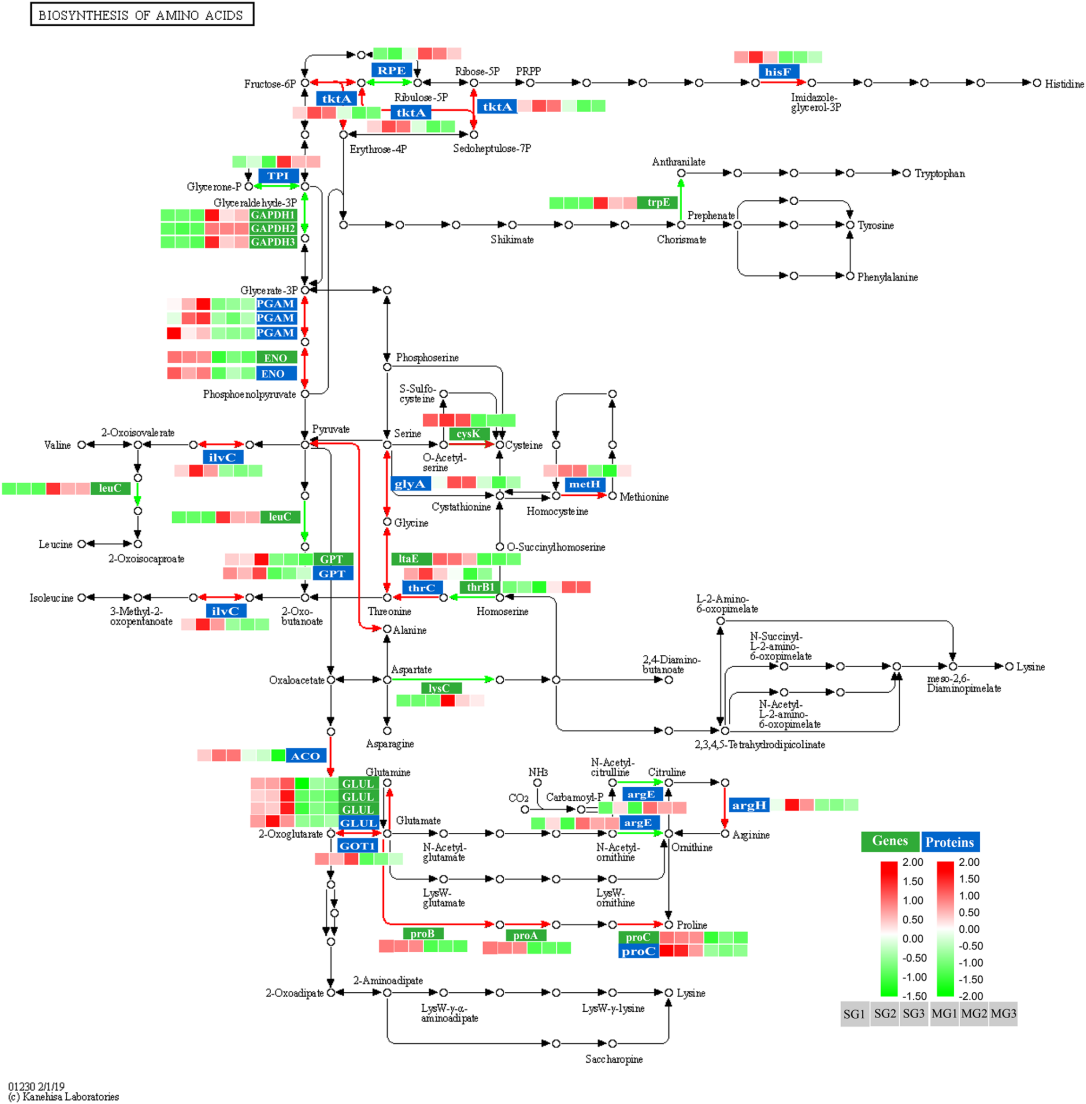
Abundance patterns of transcripts and protein species involved in the biosynthesis of amino acids of NIL-723 and NIL-1010. White characters with a green background represent genes, whereas white characters with a blue background represent protein species. For each gene or protein, the blocks represent expression levels in replications 1, 2, 3 of SG and MG from left to right. The expression value was log^2^ transformed. Red represents up-regulated and green represents down-regulated. The KEGG pathway was obtained from the Kanehisa Laboratory (www.kegg.jp/kegg/kegg1.html).

In the present study, a comprehensive investigation of changes in the amino acid synthesis pathways (www.kegg.jpkegg/kegg1.html) of DEGs and DEPs involving cultivars NIL-723 and NIL-1010 was conducted (Fig. 6). DEPs and DEGs were mainly located in the serine, cysteine, methionine, glycine, threonine, glutamic acid, arginine, and proline synthetic. Notably, most DEPs and DEGs are upregulated in the synthetic of glycine (Gly), methionine (Met), threonine (Thr), glutamic acid (Glu), proline (proC), cysteine (Cys), and arginine (Arg). 2,3-Bisphosphoglycerate-dependent phosphoglycerate mutase (PGAM) catalyzes the conversion of glycerate-3P to 2-phospho-D-glycerate, which in turn is converted to phosphoenolpyruvate through catalysis by enolase (ENO). Pyruvate is converted into alanine through catalysis by alanine transaminase (GPT). Enolase (ENO) and alanine transaminase (GPT) were upregulated in transcription and protein levels. L-homoserine inhibits the synthesis of O-phospho-L-homoserine via homoserine kinase (thrB). Additionally, threonine synthase (thrC) catalyzes the synthesis of threonine, which in turn is used to synthesize glycine through catalysis by threonine aldolase (ItaE), and serine is catalyzed to synthesize glycine through glycine hydroxymethyltransferase (glyA). HMW-GSs with high proline and glycine contents and low lysine levels have abnormally high glutamic acid content. In addition, cysteine is synthesized from O-acetylserine by cysteine synthase (cysK). Cysteine residues form intermolecular disulfide bonds between HMW-GS and LMW-GS, forming protein polymers of different sizes. Furthermore, aconitate hydratase (ACO) catalyzes the conversion of oxaloacetate to 2-oxoglutarate, and aspartate aminotransferase (GOT1) also catalyzes the conversion of 2-oxoglutarate to glutamate. GLUL encodes glutamine synthetase (GS), which catalyzes the conversion of glutamate to glutamine. ProB, ProA, and ProC are upregulated during the synthesis of Phe, and glutamic acid is involved in the biosynthesis of Phe through catalysis by glutamate 5-kinase, glutamate-5-semialdehyde dehydrogenase, and pyrroline-5-carboxylate reductase. The genes and proteins of ProC and GLUL were all upregulated in the process of proline and glutamine synthesis respectively. Besides, some DEPs or DEGs were downregulated during the synthesis of tryptophan (trpE), leucine (leuC), citrulline (argE), and ornithine (argE).

### Wheat quality-related DEGs and DEPs in protein synthesis and processing

**Figure 7:**
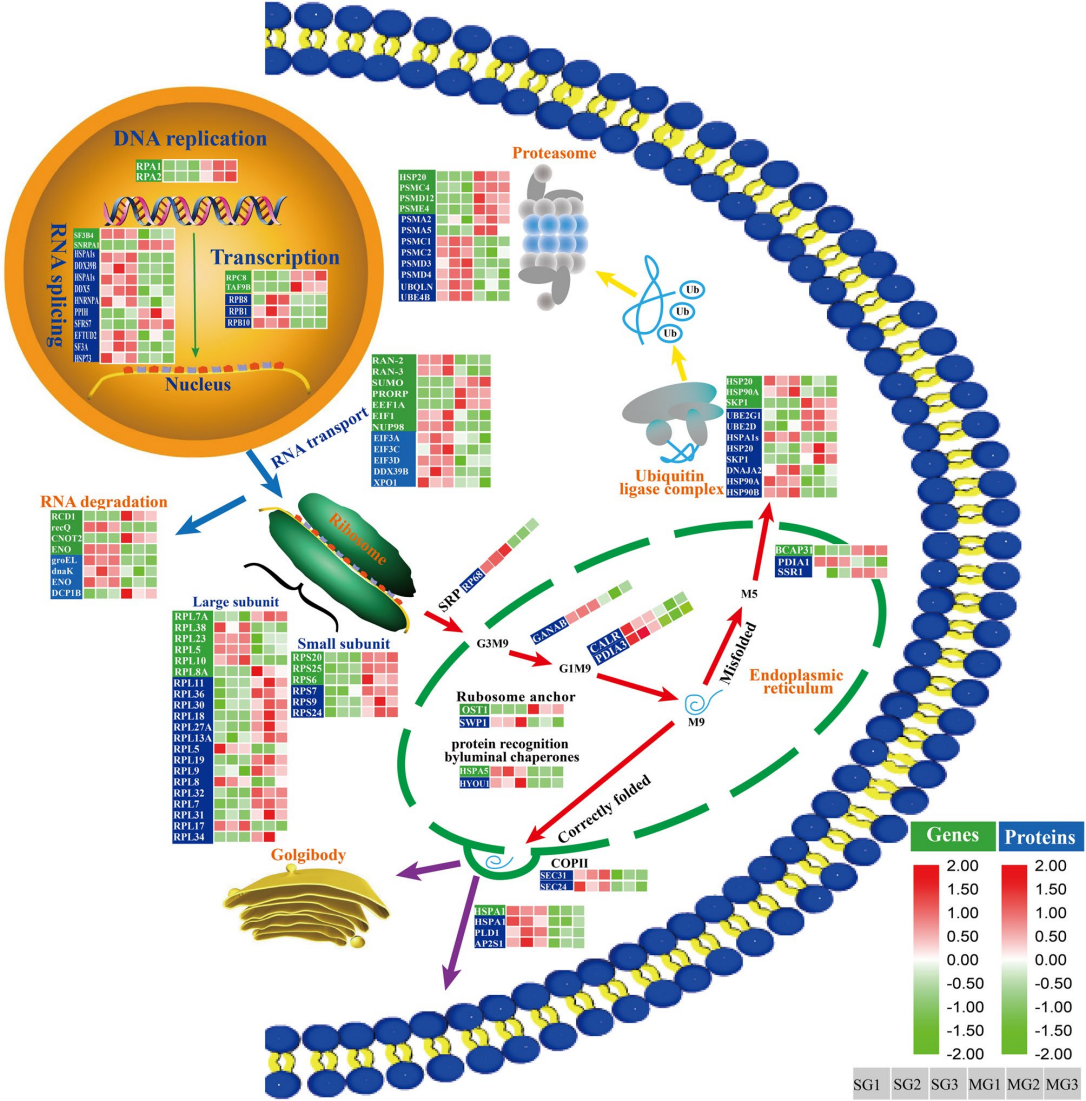
Abundance patterns of transcripts and protein species involved in protein assembly and processing of NIL-723 and NIL-1010. White characters with a green background represent genes, whereas white characters with a blue background represent protein species. For each gene or protein, the blocks represent expression levels in replications 1, 2, 3 of SG and MG from left to right. The expression value was log^2^ transformed. Red represents up-regulated and green represents down-regulated. The whole path was drawed by software Adobe illustrator (AI, CC 2019, https://www.adobe.com).

A total of three and ten DEPs were mapped to the pathways of transcription and RNA splicing, respectively (Fig. 7). Interestingly, they were all up-regulated in the grains of NIL-723, excluding two protein species (PPIH and SFRS7). Moreover, six DEGs were found to be involved in DNA replication, RNA splicing, and transcription. The transcripts of four DEGs (*RPA1*, *RPA2*, *RPC8*, and *TAF9B*) for DNA replication and transcription had reduced abundances in NIL-723 grains. In the pathway of RNA splicing, *SNRPA1* was downregulated, and *SF3B4* was upregulated in NIL-723. Five DEPs for RNA transport, namely, EIF3A, EIF3C, EIF3D, DDX39B, and XPO1, were up-regulated in NIL-723 (Fig. 7). Moreover, four DEPs and four differential transcripts were found to be involved in RNA degradation, and they were all up-regulated in NIL-723, excluding two DEGs and one protein species (*RCD1*, *CNOT2*, and DCP1B) (Fig. 7). A total of 18 ribosome-related DEPs were detected, including 15 large subunit protein species and 3 small subunit protein species. Significantly, all of these protein species were down-regulated in NIL-723, excluding three protein species (RPL5, RPL8, and RPL17) (Fig. 7). Besides, nine DEGs were also to be involved in ribosome assembly. Similarly, the five DEGs were downregulated in NIL-723, while *RPL38*, *RPL23*, *RPL5*, and *RPL10* up-regulated in NIL-723 (Fig. 7).

Endoplasmic reticulum (ER) is a multifunctional organelle that plays a vital role in protein synthesis, folding, output, and maturation. In the category ER protein processing, three differentially expressed genes were identified (OST1 and BCAP31 downregulated and HSPA5 upregulated), and nine DEPs were detected (Fig. 7). Except for SSR1, the other eight DEPs were significantly upregulated in NIL-723, and the upregulation of the ribosome-anchored subunit SWP1 could promote the glycosylation of proteins and the developmental phenotype of grains. The upregulation of the endoplasmic reticulum (ER) homologs HSPA5 and HYOU1 (BiP) regulates protein synthesis through complex interactions with various substrates and regulatory proteins. The GANAB of the alpha subunit of glucosidase II plays a critical role in protein folding and quality control and is recognized by lectin-chaperones, calnexin, and calreticulin. PDIA3 and PDIA1 were upregulated in NIL-723, indicating that they played a positive regulatory role in the synthesis of gluten. CALR is a CRT family of proteins responsible for regulating calcium homeostasis and promoting the folding and synthesis of gluten with Hsp70 and PDI in the endoplasmic reticulum. Secretory24 (Sec24) and secretory31 (Sec31) upregulated in NIL-723 promote the anterograde transport of newly synthesized proteins from the endoplasmic reticulum (ER) to other compartments in the plant endometrium mediated by shell protein complex II (COPII). Heat shock 70 kDa protein (HSPA1s) and phospholipase D1(PLD1) detected and upregulated in NIL-723 were found to be essential for the ER stress response.

Protein ubiquitination plays a crucial role in regulating the post-transcriptional modification of proteins. In the ubiquitin ligase complex, eight DEPs (UBE2G1, UBE2D, HSPA1s, HSP20, HSP90A, HSP90B, SKP1, and DNAJA2) and three DEGs (*HSP20*, *HSP90A*, and *SKP1*) were identified. The protein species HSP20, SKP1, UBE2G1, and UBE2D and the transcripts of *SKP1* were down-regulated, whereas the transcripts of *HSP20*, *HSP90A*, and DEPs of HSPA1s, HSP90A, HSP90B, and DNAJA2 were up-regulated in NIL-723 (Fig. 7). Moreover, eight DEPs and four DEGs were mapped to phagolysosomes that responded differently to gluten synthesis, six of them were down-regulated, and the rest were up-regulated in NIL-723 (Fig. 7).

### Validation of differentially expressed proteins at the transcription level

**Figure 8:**
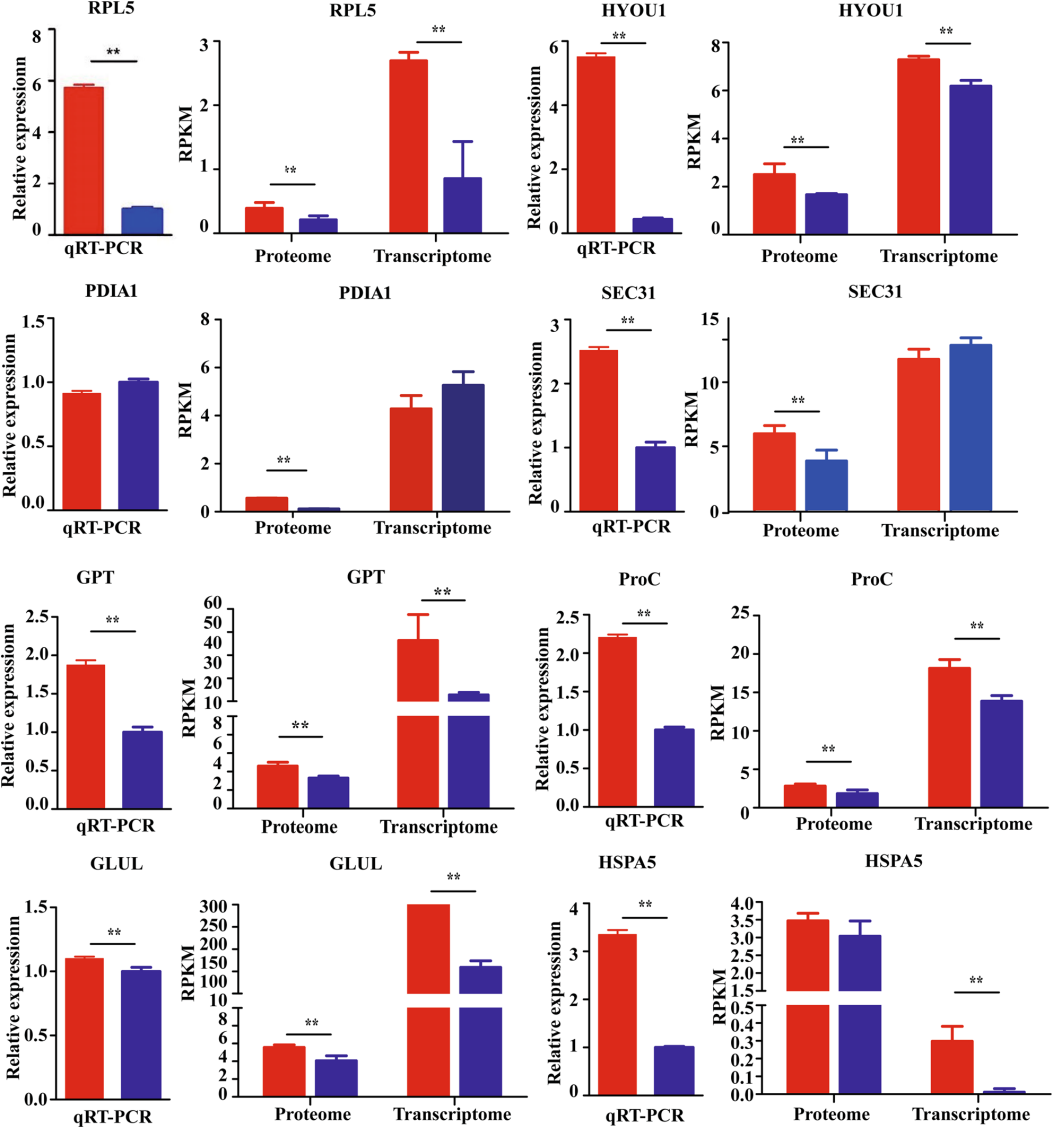
Relative transcript expression of selected differentially expressed proteins and genes identified through combined analysis. Relative expression of each gene was normalized with the actin signal. Red bars represent NIL-723, and blue bars represent NIL-1010. RPL5, ribosomal protein L5; HYOU1, hypoxia up-regulated 1; PDIA1, Protein disulfide-isomerase A1; SEC31, COPII coat complex component secretory31; GPT, glutamic-pyruvic transaminase; ProC, proline C; GLUL, glutamate-ammonia ligase; HSPA5, heat shock 70 kDa protein 5. Bars indicate means ± standard deviations (SDs) of at least three independent biological replicates. Two asterisks indicate a significant difference at *p* < 0:01. Statistical analysis was performed with SPSS Statistics 26.0 software (https://www.ibm.com/products/spss-statistics).

The transcript abundance of some of the differentially abundant proteins and genes was validated through the transcript abundance of their corresponding genes using quantitative real-time PCR (RT-qPCR). The RT-qPCR results corroborated that the relative abundance at the transcript level was consistent with the proteomic and transcriptome analysis (Fig. 8, Table S10). However, the transcript level of some of the proteins was found to be completely opposite to that of the differential expression of their genes. The relative expression of PDIA1 was found to be insignificant, whereas the relative abundance of transcription was extremely significant. The relative abundance of RPL5, HYOU1, and HSPA5 increased significantly at the transcriptional and proteomic levels of NIL-723, which were also verified by quantitative real-time PCR. We think that these three candidate genes are related to the quality of wheat and can be used as the basis for transgenic or molecular marker-assisted selection to improve the quality of wheat. We will conduct the genetic transformation of the target gene in wheat for further verification.

## Discussion

Wheat quality is substantially significant to the standard living of individuals^1^. We previously reported many HMW and LMW glutenin alleles, but the mechanisms by which these genes regulate the wheat glutenin synthesis pathway is unclear. This study aimed to investigate the potential regulatory mechanisms involved in the superior cultivar NIL-723. To this end, we collected grains of the near-isogenic lines NIL-723 and NIL-1010 during the grain filling stage for transcriptomic and proteomic analyses. Compared with NIL-1010, a total of 1287 transcripts and 355 protein species in NIL-723 had significantly different abundance in the transcriptome and proteome, respectively. Theoretically, these changes in abundance led to the higher quality of NIL-723 compared with NIL-1010. Generally, the process from mRNA to protein is highly complicated. The abundance of protein species is usually affected by complex regulatory processes, such as DNA methylation, non-coding RNA interactions, post-translational modification, mutation, degradation, and protein interactions^28^. Therefore, the R programming language was used to calculate the Pearson correlation coefficient, and the consistency between changes in transcript and protein species abundance between NIL-723 and NIL-1010 was studied. Changes in 2128 genes in the transcriptome were consistent with the proteome, while 384 and 3 molecules exhibited significant differences and opposite changes in the transcriptome and proteome, respectively, indicating that strong post-translational regulation in the synthetic process from mRNA to protein may be involved. We also analyzed the protein interaction network of the top 100 DEGs and DEPs and found that approximately a third of the proteins were significantly downregulated and that two-thirds were significantly upregulated. Most of these proteins are associated with starch and protein synthesis and assembly, indicating that the excellent quality of NIL-723 may be affected by starch and protein synthesis.

Studies have confirmed that amino acids are closely associated with glutenin synthesis^5^. DEPs and DEGs in amino acid synthesis pathways are mainly located in the synthetic pathways of serine, cysteine, methionine, glycine, threonine, glutamic acid, arginine, and proline. Most DEPs or DEGs were upregulated in the synthetic pathways of glycine, methionine, threonine, glutamic acid, proline, cysteine, and arginine. This is consistent with the finding of high glutamic acid, proline, and glycine content and low lysine levels in HMW-GS^33^. The genes and proteins of the GLUL and ProC family proteases were upregulated to catalyze the biosynthesis of glutamine and phenylalanine, respectively. In addition, the accumulation of cysteine residues promotes the formation of intermolecular disulfide bonds between HMW-GS and LMW-GS and folding into the protein polymers of different sizes^11^.

In this study, we plotted a regulatory pathway map to elucidate changes in glutenin in the grain synthesis pathway. Eleven DEPs in transcription and RNA splicing pathways were upregulated in NIL-723 grains, and one DEG (SF3B4) in RNA splicing pathways was upregulated. SF3B4 is a transcription factor for RNA splicing and a cofactor that binds to proteins on the endoplasmic reticulum (ER) that plays a key role in splicing and enhancing the translation of specific proteins in plants^34^. A total of 18 DEPs and 9 DEGs associated with ribosomes were detected, of which only 3 proteins (RPL5, RPL8, and RPL17) and 4 genes (*RPL5, RPL10, RPL23*, and *RPL38*) were upregulated in NIL-723. The RPL5 transcript and protein were upregulated in the ribosomes of NIL-723, and RP protein mediates selective translation^35^, suggesting that RPL5 may be necessary for the translation of specific glutenins. EIF3 is the most complex translation initiation factor and plays a role in translation elongation and enhances the synthesis of proteins associated with membrane function in protein synthesis^36^. EIF3. A total of nine DEPs and three DEGs associated with ER protein processing were detected. SWP1, an anchor subunit of ribosomes, is mutated in Arabidopsis with reduced protein glycosylation and severe dysplasia^37^. HSPA5 and HYOU1 (BiP) are the homologous proteins of the Hsp70 family of chaperone proteins in the ER, which are involved in most functions of protein synthesis by complex interactions with multiple substrates and regulatory proteins, such as assisting folding, assembly and disassembly of protein complexes, pulling polypeptides from ribosomes and transmembrane pores, activating and inactivating signaling proteins and controlling their degradation^38^. Zhu et al.^39^ found that the expression level of BiP is associated with the subunit type and synthesis of HMW-GS. GANAB, the alpha subunit of glucosidase II, plays a critical role in protein folding and quality control and is recognized by lectin-chaperones, calnexin, and calreticulin^40^. Kimura et al.^41^ argued that protein disulfide isomerase (PDI) family proteins in the ER of wheat endosperm cells play an important role in gluten synthesis and folding during grain filling. The upregulation of PDIA3 and PDIA1 in NIL-723 indicates that they play a positive regulatory role in gluten protein synthesis. At the same time, CALR is a CRT family protein that regulates calcium homeostasis and can promote gluten protein folding and synthesis in the ER via Hsp70 and PDI. Additionally, Wang et al.^19^ validated that Sec24 is associated with the accumulation of glutenin precursors and plays an essential role in mediating COPII vesicle formation and promoting the transport of secreted proteins in plant cells. The present study found that Sec24 and Sec31 are upregulated in NIL-723.

The genes associated with wheat quality found in the present study provide a reference for the artificial regulation of wheat quality using genetic engineering techniques, which may have potential applications in agriculture. However, the functions of most DEPs or DEGs found in the present study are still largely unknown, and we will continue to investigate the function of these genes.

## Conclusions

The combined analysis of the transcriptome and proteome produced a more complete regulatory map for the wheat glutenin synthesis pathway, which has deepened our understanding of wheat glutenin synthesis and the networks involved. In addition, genes related to wheat quality identified in this study provide a reference for future studies, and genetic engineering techniques could be used to artificially regulate wheat quality, which may have potential applications in agriculture.

## Materials and methods

### Plant materials

The near-isogenic lines (NILs) were generated by backcrossing Taigu genomic sterile population and high-quality cultivar Shiluan 02–1 for six generations, followed by the three generations of selfing to homozygosity. The near-isogenic lines NIL-723 and NIL-1010, which had the same agronomic traits and gliadins, HMW-GS except for the difference in LMW-GS, were provided by the Institute of Cereal and Oil Crops, Hebei Academy of Agriculture and Forestry Sciences, Shijiazhuang, China. The NIL-723 has superior bread quality, with longer stability time and greater the maximum tensile resistance. In June 2017, the seeds were planted in randomized complete blocks with three replicates at an experimental base of Shijiazhuang, Hebei Province, China (114.84°, 37.59° E N, 50 m). The management conditions and techniques of the field were consistent. At 25 days after flowering, five wheat plants with the same growth vigor were selected from each material in each repetition, and the central part grains of the main spike were collected for transcriptomic and proteomic analyses. The prepared samples and seeds were crushed in liquid nitrogen to avoid tissue oxidation. All experiments were repeated thrice. The present study complies with relevant institutional, national, and international guidelines and legislation.

### Flour characteristics

After harvest, divergent varieties of grains were conditioned for 24 h at 14% moisture and milled in the Quadrumat Senior Mill (Barbender, Germany). According to the AACC (American Association for Clinical Chemistry 2000) method, the dough properties were determined using a farinograph (Brabender, Germany). The water absorption (WA) was determined as the amount of water required to center the farinograph curve at 500 BU. Dough development time (DDT) is defined as the time between the first addition of water to the dough to its maximum consistency. Dough stability (DS) was the time difference between the first intercept of the top of the curve at 500 BU and the departure of the top of the curve at 500 BU. The protein, sedimentation value, and wet gluten of flour were determined by employing the standard method (AACC 2000). Each sample was repeated three times in the analysis of flour quality.

### Transcriptome sequencing

The total RNA was extracted from all the obtained samples using the RNA Prep Pure Plant Kit (TIANGEN, Beijing, China) according to the manufacturer’s instructions. The library construction and sequencing of six RNA samples were completed by Novogene Technology Co. Ltd. (Beijing, China). The RNA-Seq data were submitted to the Sequence Read Archive (SRA) with the accession number PRJNA615781. After the raw sequences were filtered, clean reads were aligned against the *Triticum aestivum* genome (http://plants.ensembl.org/Triticum_aestivum/Info/Index) using HISAT2 (v2.1.0)^42,43^. The gene expression level of RNA-Seq was estimated by the reads per kilobase per million mapped read (RPKM)^44^. DESeq2 (v1.26) was employed to analyze the differential gene expression^45^, and genes with *p* < 0.05 and |log2 ratio|> 1 were identified as differentially expressed genes.

### Data processing for proteomics

The samples were ground into powder using a mortar and transferred to a 1.5-ml screw-capped tube. Each sample, after adding 100 μl of lysis buffer (7 M urea, 2 M sulfourea, 0.1% CHAPS, 1 × protease inhibitors), was ultrasonically crushed to extract the total protein and centrifuged at 14,000 × *g* at 4 °C for 30 min. The supernatant was used for further experiments. With a Pierce BCA protein assay kit (Thermo Fisher Scientific Inc.) using bovine serum albumin (BSA) as a standard, protein concentration was determined at a wavelength of 595 nm. The standard proteins were sequentially added to 96-well microtiter plates, followed by the addition of pure water, and 180 μl of Coomassie Brilliant Blue to calculate the protein concentration of each sample. Protein (350 ug) was loaded on 24 cm IPG strip with a linear gradient (pH 4–7), and 2D gel electrophoresis of SDS-PAGE was performed with 12.5% gel^46^. After SDS-PAGE electrophoresis, the proteins were stained for 2 h with 0.05% Coomassie Brilliant Blue (Sigma, USA) and then with a decoloring solution. After completely destaining and washing, Imagemaster 2D Platinum Software Version 5.0 (GE Healthcare, USA) was used to detect spots with the parameters smooth, minimum area and saliency set to 2, 15 and 8, respectively, followed by manual spot editing, such as artificial spot deletion, splitting and merging. The tryptic digestion and in-gel digestion of gel pieces were performed according to the method previously described by Shevchenko et al.^47^. The digested peptides were separated using Thermo Scientific EASY-nLC 1000 system (Nano HPLC) and detected by the Orbitrap Fusion mass spectrometer (Thermo Scientific)^46^. The full-scan mass spectrometry was obtained in the mass spectrometry over 350–1800 m/z with a resolution of 700,000 and a spectral resolution of 17,500, and the normalized collision energy was set to 29%.

The MS raw data were processed using the MaxQuant software (Max-Planck-Institute of Biochemistry, Martinsried, Germany, https://www.maxquant.org). The MS data were searched against the wheat genome database (http://plants.ensembl.org/Triticum_aestivum/Info/Index). The parameters of MaxQuant searches were set as follows: carbamidomethylation (C) and oxidization (M) were set as static and dynamic modifications, respectively; precursor and fragment ion mass tolerance were 15 ppm and 20 mmu, respectively; and max missed cleavages with ≤ 2 were permitted. The threshold of the global false discovery rate (FDR) for peptides and proteins was set to 0.01. After protein identification, the peptide signal intensities were used to calculate the strength of each identified protein. According to the MaxLFQ label-free quantification method, retention time alignment, label-free quantification, and MaxLFQ normalization were performed as previously described^48^. The identification transfer protocol was performed in the experimental replication to extract quantitative information across the replication. The peak intensity from the complete set of measurements was compared by Perseus to obtain quantitative data for all peptides in the sample (version 1.4.1.3). The LFQ (label-free quantification) protein intensity obtained through MaxQuant analysis was introduced and converted to a two-based logarithmic scale. The lowest intensity value is used to replace the missing value to compensate for the low signal of low abundance protein. A two-way Student’s t-test was used for protein quantitative and statistical significance analysis. Protein species with fold-change > 1.5 and FDR-adjusted (*p*-value < 0.05) were identified as differentially expressed protein (DEPs) between the experimental groups. FDR-adjusted (*p* < 0.05) was as corrected by the Benjamini–Hochberg procedure, and the mass spectrometry proteomics data were deposited to the ProteomeXchange Consortium (http://proteomecentral.proteomexchange.org) via the iProX partner repository with the dataset identifier PXD020200.

### Bioinformatics analysis

The bioinformatics analysis of protein species was performed according to Lin et al.^27^, and the biological processes, molecular functions, and cellular components of DEGs and DEPs were analyzed using Blast2GO (version 5.2) (http://www.blast2go.com)^49^. Moreover, DEGs and DEPs were investigated using the Kyoto Encyclopedia of Genes and Genomes (KEGG) to map to the possible KEGG pathway maps for the biological interpretation of systemic functions (https://www.kegg.jp/)^50^. Protein–protein interactions were obtained from the Search Tool for the Retrieval of Interacting Genes/Proteins (STRING) database (http://string-db.org/) containing known and predicted physical and functional protein–protein interactions^51^. The multiple sequence alignment was performed using MAFFT (v7.450)^52^.

### Quantitative real-time PCR (RT-qPCR)

According to the manufacturers’ instructions, total RNA was isolated using the Trizol reagent (Invitrogen, Grand Island, NY, USA). The first cDNA was synthesized by the M-MLV reverse transcriptase (Takara, Tokyo, Japan). The Primer Premier 6.0 (Premier Biosoft International, Palo Alto, CA, USA) was used to design specific primers. The reaction was carried out in 20 µL containing 1 µL cDNA, 10 mM Tris–HCl (pH 8.5), 50 mM KCl, 2 mM MgCl2, 0.4 µL of DMSO, 200 mM dNTPs, 10 pmol/µL specific PCR primers, 1 U of Taq DNA polymerase and 0.5 µL of SYBR GREEN I fluorescence dye. The RT-qPCR was conducted in clear tubes using an Applied Biosystems ViiATM 7 Real-Time PCR System (Carlsbad City, CA, USA) as follows: 94 °C for 5 min, 40 cycles at 94 °C for 30 s, 58 °C for 30 s and 72 °C for 45 s, and a final extension at 72 °C for 5 min. Actin as an internal control was used to normalized the mRNA expression level^53^. The actin expression was stable and did not change across all of the RNAseq datasets. Three biological replicates of RT-qPCR were performed for each sample. The average values of 2^−△Ct^ were used to identify differences in gene expression^54^.

### Statistical analysis

Statistical analysis was performed with SPSS Statistics 26.0 software (https://www.ibm.com/products/spss-statistics). One-way analysis of variance(ANOVA) was used to evaluate the significance of differences between NIL-723 and NIL-1010. Experimental data was represented by the mean ± standard deviation (SD). The Student’s t-test was used to evaluate the significance of the differences among NILs. In all cases, *p* < 0.05 was indicated statistically significant. Unsupervised PCA (principal component analysis) was performed by statistics function prcomp within R 3.5.0 (www.r-project.org)^31,32^. The data was unit variance scaled before unsupervised PCA.

## Supplementary information

Supplementary Information 1.

Supplementary Information 2.

Supplementary Information 3.

Supplementary Information 4.

Supplementary Information 5.

Supplementary Information 6.

Supplementary Information 7.

Supplementary Information 8.

Supplementary Information 9.

Supplementary Information 10.
